# Antimicrobial and antibiofilm activities of Cu(II) Schiff base complexes against methicillin-susceptible and resistant *Staphylococcus aureus*

**DOI:** 10.1186/s12941-021-00473-4

**Published:** 2021-09-24

**Authors:** Pooi Yin Chung, Ranon Earn Yueh Khoo, Hui Shan Liew, May Lee Low

**Affiliations:** 1 Department of Microbiology, School of Medicine, International Medical University, Kuala Lumpur, Malaysia; 2 School of Postgraduate Studies and Research, International Medical University, Kuala Lumpur, Malaysia; 3 Department of Pharmaceutical Chemistry, School of Pharmacy, International Medical University, Kuala Lumpur, Malaysia; 4 School of Medicine, International Medical University, Kuala Lumpur, Malaysia

**Keywords:** Schiff base derivatives, Biofilms, Methicillin-resistant *Staphylococcus aureus*

## Abstract

**Background:**

Methicillin-resistance *S. aureus* (MRSA) possesses the ability to resist multiple antibiotics and form biofilm. Currently, vancomycin remains the last drug of choice for treatment of MRSA infection. The emergence of vancomycin-resistant *S. aureus* (VRSA) has necessitated the development of new therapeutic agents against MRSA. In this study, the antimicrobial and antibiofilm activities of two copper-complexes derived from Schiff base (SBDs) were tested individually, and in combination with oxacillin (OXA) and vancomycin (VAN) against reference strains methicillin-susceptible and methicillin-resistant *Staphylococcus aureus*. The toxicity of the SBDs was also evaluated on a non-cancerous mammalian cell line.

**Methods:**

The antimicrobial activity was tested against the planktonic *S. aureus* cells using the microdilution broth assay, while the antibiofilm activity were evaluated using the crystal violet and resazurin assays. The cytotoxicity of the SBDs was assessed on MRC5 (normal lung tissue), using the MTT assay.

**Results:**

The individual SBDs showed significant reduction of biomass and metabolic activity in both *S. aureus* strains. Combinations of the SBDs with OXA and VAN were mainly additive against the planktonic cells and cells in the biofilm. Both the compounds showed moderate toxicity against the MRC5 cell line. The selectivity index suggested that the compounds were more cytotoxic to *S. aureus* than the normal cells.

**Conclusion:**

Both the SBD compounds demonstrated promising antimicrobial and antibiofilm activities and have the potential to be further developed as an antimicrobial agent against infections caused by MRSA.

**Supplementary Information:**

The online version contains supplementary material available at 10.1186/s12941-021-00473-4.

## Background

*Staphylococcus aureus* has been recognized as a prominent human pathogen that causes complications ranging from minor to life-threatening infections. Of particular interest are infections associated with catheters and other indwelling medical devices which are characterized by biofilm formation [1]. The bacteria within the biofilms are embedded in a matrix of exopolysaccharides glycocalyx. This matrix protects the enclosed bacteria from host defenses (phagocytosis) and limits the diffusion of antibiotics [2]. In addition, multidrug-resistant isolates of methicillin-resistant *Staphylococcus aureus* (MRSA) exhibiting decreased susceptibilities to glycopeptides has also emerged, representing a crucial challenge for antimicrobial therapy and infection control [3]. Therefore, there is an urgent need to develop antibacterial agents with novel mechanisms of action and higher activity to address the multidrug resistance challenge and biofilm formation. One of the strategies is to screen and discover chemical compounds that are foreign to the bacteria such as the Schiff base complexes.

The coordination chemistry of Schiff base chelating ligands provides a rich platform for the design of metallodrugs. Schiff bases are condensation products of primary amines and aldehydes or ketones (RCH = NR’, where R and R’ represents alkyl and/or aryl substituents) that have often been used in the preparation of complexes. They can form open chains and macrocyclic/macroacyclic ligands as well as mixed ligand system with varying denticity and nucleating abilities [4]. In the attractive area of bioinorganic chemistry, interest in Schiff base complexes has centered on the role such complexes may have as potential therapeutic drug candidates, diagnostic agents and in providing synthetic models for the metal containing sites in metalloproteins and metalloenzymes [5]. In particular, the Schiff base derivatives have been shown previously to be active against *S*. *aureus* [6]. Based on these considerations, this study focused on the antimicrobial and antibiofilm activities of Schiff base derivatives against multidrug-resistant and biofilm-forming *S. aureus* utilizing the concept of synergistic effect with antibiotics that is anticipated to result in potential metallodrug candidates having pronounced biological activity with enhanced selectivity.

## Materials and methods

### Bacteria strains, antibiotics and Cu(II) Schiff base complexes

Reference strains of *S. aureus* ATCC 29213 and ATCC 43300 representing methicillin-susceptible (SA) and methicillin-resistant *S. aureus* (MRSA), respectively, and a non-cancerous human cell line MRC5 (normal lung tissue) were used in the study. Two Cu(II) Schiff base complexes (SBD2 and SBD4) which have been prepared previously were also used (Table 1). Copper was chosen to complex with the SBD ligands as it was known to increase the potency [7]. Vancomycin (VAN) and oxacillin (OXA) which were used as controls to compare the antimicrobial and antibiofilm activities were obtained from Sigma-Aldrich.

**Table.1:** Cu(II) Schiff base complexes used in the study

Name	Compound description	Molecular structure	Molecular weight (g/mol)
SBD2	Cu(SB4CB)_2_: Copper complex of SB4CB		722.37
SBD4	Cu(SBFH)_2_: Copper complex of SBFH		754.37

### Preparation of antibiotics and compounds

The stock solutions of the SBD compounds and antibiotics were prepared in a concentration of 5 mg/mL by dissolving the solid powder in 100% dimethyl sulfoxide (DMSO), and ultrapure water, respectively. The stock solutions were then serially diluted two-fold to obtain concentrations in the range of 1 to 128 µg/mL (Additional file 1: Fig. S1).

### Antimicrobial assays: microdilution broth assay

The antimicrobial activity of the two Cu(II) Schiff base complexes and antibiotics were determined using the microdilution broth assay [8] with a final inoculum of 1 × 10^5^ cfu/mL. Tryptic soy broth (TSB, Merck) was optimized as the media for the assay (Additional file 5: Table S1). Each concentration was performed thrice in five replicates (n = 5 × 3). VAN at 4 μg/mL and OXA at 16 μg/mL were used as positive controls while DMSO diluent at 5% and TSB were the negative controls. The plates were then incubated at 37 °C for 24 h. The lowest concentration of SBD compounds with clear wells were recorded as the minimum inhibitory concentration (MIC). Every concentration which showed clear wells were then cultured on tryptic soy agar (TSA, Merck) plates in five replicates. After an incubation for 24 h at 37 °C, the lowest concentration of SBD compound that showed no growth of bacteria colonies were recorded as the minimum bactericidal concentration (MBC).

The sets of combinations between the SBD compounds and the antibiotics in concentrations ranging from 0.0625 × to 1 × MIC were generated using the checkerboard method. Fractional Inhibitory Concentration (FIC) index was used to interpret the interaction of every combination that showed inhibition of bacterial growth. The FIC index for each compound in the combinations was calculated as follows:

$${\text{FIC}}\;{\text{index}} = \frac{{{\text{MIC}}\;{\text{of}}\;{\text{SBD}} \times {\text{in}}\;{\text{combination}}}}{{{\text{MIC}}\;{\text{of}}\;{\text{SBD}} \times {\text{alone}}}} + \frac{{{\text{MIC}}\;{\text{of}}\;{\text{VAN/OXA}}\;{\text{in}}\;{\text{combination}}}}{{{\text{MIC}}\;{\text{of}}\;{\text{VAN/OXA}}\;{\text{alone}}}}$$

A FIC index of ≤ 0.5 was considered synergistic where the combined effect of both agents is more effective than single agents. An index of 0.5 to 4 was additive where the interaction of both agents was mutually exclusive. The interaction was antagonistic, where one agent counteracts the action of the other agents, reducing the efficacy when the index was higher than 4 [9].

### Biofilm formation of SA and MRSA

Biofilms were formed by the addition of bacterial suspension in TSB with an estimated inoculum of 1 × 10^5^ cfu/mL in sterile, flat-bottomed, 96-well microtiter plates (Thermo Fisher Scientific, Denmark). The plates were then incubated for 20 h under static and aerobic conditions for mature biofilms to form.

### Anti-biofilm assays: crystal violet (CV) and resazurin (RZ) assays

The two SBD compounds were also evaluated for their anti-biofilm activities. The eradication of the biomass of the biofilm formed by SA and MRSA was evaluated using the modified 96-well microtiter plates assay [10]. The preformed (mature) biofilms in the wells of the microtiter plate were treated with the SBD compounds and antibiotics (OXA and VAN). The control wells were similar to the antimicrobial assay. The microtiter plate was then incubated for 24 h at 37 °C under static and aerobic conditions. The culture supernatant and non-adherent bacteria were then removed by decanting and washing the wells three times with phosphate buffered saline (PBS). The biofilms were stained with 0.1% (v/v) CV, fixed with methanol at room temperature for 15 min and washed again three times with PBS to rid the plate of all excess cells and dye, and dried. The CV stain were released from the adherent cells with 30% acetic acid and the microtiter plate were incubated at room temperature for 5 min. The solubilized CV stain was then transferred to a new microtiter plate. The absorbance was quantified at 570 nm at specific time-points over 24 h. The blank control used was 30% acetic acid in water.

The metabolic activity, an indicator of cell viability, was measured using the RZ stain. In this assay, pre-formed biofilms of MRSA were washed with phosphate buffered saline (PBS), after which 50 µL of 0.01% (v/v) aqueous RZ (Acros Organics, Belgium) solution was added. The microtiter plates were then incubated in the dark under static and aerobic conditions at 37 °C for 3 h. Fluorescence intensity was measured at 560/590 nm using a microplate reader at time-points similar to that of the CV assay. The blank control used to correct for background signal was sterile TSB.

Both the CV and RZ stain assays were performed in triplicates and repeated three times (n = 3 × 3). The lowest concentration of OXA, VAN and SBD compounds that significantly reduce the biomass of the biofilm was recorded as the minimum biofilm eradication concentration (MBEC).

The combination effects of the SBD compounds and OXA or VAN on the biomass of MRSA were evaluated at 0.25 × to 1 × MBEC, using the checkerboard method. The fractional biofilm eradication concentration (FBEC) of each combination was calculated and interpreted using a similar formula as the FIC index. The experiments were performed in triplicates and repeated three times (n = 3 × 3).

### Cytotoxicity assay

A non-cancerous human cell line MRC5 (normal lung tissue) were cultured in Roswell Park Memorial Institute Medium (RPMI) 1640 supplemented with 10% foetal bovine serum, and 1% penicillin and streptomycin. The cells were seeded in a 96-well flat-bottom microtiter plate at a density of 5 × 10^3^ cells per well and allowed to adhere for 24 h at 37 °C in a 5% CO_2_ humidified incubator. After replacing the culture medium with a fresh medium, the cells were then treated with the SBD compounds at two-fold concentrations ranging from 1 to 256 µg/mL and incubated under similar conditions. Subsequently, 25 µL of MTT [3-(4,5-dimethylthiazole-2-yl)-2,5-diphenyltetrazolium bromide] solution (5 mg/mL in phosphate buffer solution) was added to each well and the plate was incubated for 4 h at 37 °C in a 5% CO_2_ humidified incubator until the purple precipitate formazan was visible. The precipitate was solubilized by adding 100 µL of DMSO/glycine (4:1) in each well and left at room temperature in the dark for 2 h. The intensity of the dissolved precipitate was quantified at 570 nm. The concentration of each of the compounds that caused 50% inhibition of cell viability (IC_50_) and percentage of cell viability were determined. The selectivity index (SI) values were calculated by cytotoxicity IC_50_ values and MIC values (IC_50_/MIC) [11]. The positive controls were OXA and VAN, while the negative controls were untreated cells and diluent, i.e. culture medium RPMI 1640 without the compounds and methanol, respectively. The assay was carried out in triplicates.

### Statistical analyses

All values were expressed as means ± standard deviation from replicates of the experiments. A one-way analysis of variance was used to determine the differences in biofilm formation and metabolic activity between the control (without treatment) and each test group (SPSS software version 17.0). Differences achieving a confidence level of 95% were considered significant.

## Results

### Antimicrobial activity of SBD compounds and antibiotics against planktonic SA and MRSA: individual and in combination with antibiotics

The two copper-complex derived Schiff base, SBD2 and SBD4, showed promising inhibitory activity against both SA and MRSA (Table 2). Based on the MIC values of SBD2, SBD4, OXA and VAN, 24 and 59 combinations out of a total of 100 combinations of SBD2 and SBD4 with OXA and VAN showed inhibitory activity against SA and MRSA, respectively (Tables 3, 4, 5, 6). A total of four combinations of SBD2 and OXA showed synergism against SA and MRSA while two combinations of SBD4 and OXA showed synergism against SA. In combinations of the SBD compounds and VAN against MRSA, four combinations showed synergism. Bactericidal activity was observed in six combinations of the SBD4 with OXA and VAN against SA and MRSA.

**Table.2:** MIC and MBC values of the SBD compounds and antibiotics against SA and MRSA

Compound	SA		MRSA	
	MIC (μg/mL)	MBC (μg/mL)	MIC (μg/mL)	MBC (μg/mL)
SBD2	32	> 128	8	> 128
SBD4	16	> 128	32	> 128
VAN	2	4	4	16
OXA	1	16	16	> 128

**Table 3:** Combination effect of SBD and OXA on SA

Compound	Concentration (μg/mL)		FIC index		Sum FIC index	Interaction	Static/cidal effect
	SBD	OXA	SBD	OXA			
SBD2	8	2	1.00	0.13	1.13	Additive	Bacteriostatic
	4	2	0.50	0.13	0.63	Additive	Bacteriostatic
	2	2	0.25	0.13	0.38	Synergy	Bacteriostatic
	1	2	0.13	0.13	0.26	Synergy	Bacteriostatic
	0.5	2	0.06	0.13	0.19	Synergy	Bacteriostatic
	8	1	1.00	0.06	1.06	Additive	Bacteriostatic
SBD4	32	2	1.00	0.13	1.13	Additive	Bacteriostatic
	16	2	0.50	0.13	0.63	Additive	Bacteriostatic
	8	2	0.25	0.13	0.38	Synergy	Bacteriostatic
	4	2	0.13	0.13	0.26	Synergy	Bacteriostatic
	32	1	1.00	0.06	1.06	Additive	Bacteriostatic

**Table.4:** Combination effect of SBD and OXA on MRSA

Compound	Concentration (μg/mL)		FIC index		Sum FIC index	Interaction	Static/cidal effect
	SBD	OXA	SBD	OXA			
SBD2	8	16	1.00	1.00	2.00	Additive	Bacteriostatic
	4	16	0.50	1.00	1.50	Additive	Bacteriostatic
	2	16	0.25	1.00	1.25	Additive	Bacteriostatic
	1	16	0.13	1.00	1.13	Additive	Bacteriostatic
	0.5	16	0.06	1.00	1.06	Additive	Bacteriostatic
	8	8	1.00	0.50	1.50	Additive	Bacteriostatic
	8	4	1.00	0.25	1.25	Additive	Bacteriostatic
	4	4	0.50	0.25	0.75	Additive	Bacteriostatic
	8	2	1.00	0.13	1.13	Additive	Bacteriostatic
	8	1	1.00	0.06	1.06	Additive	Bacteriostatic
	4	1	0.50	0.06	0.56	Additive	Bacteriostatic
	2	1	0.25	0.06	0.31	Synergy	Bacteriostatic
SBD4	32	16	1.00	1.00	2.00	Additive	Bactericidal
	16	16	0.50	1.00	1.50	Additive	Bacteriostatic
	8	16	0.25	1.00	1.25	Additive	Bacteriostatic
	4	16	0.13	1.00	1.13	Additive	Bacteriostatic
	2	16	0.06	1.00	1.06	Additive	Bacteriostatic
	32	8	1.00	0.50	1.50	Additive	Bactericidal
	16	8	0.50	0.5	1.00	Additive	Bacteriostatic
	32	4	1.00	0.25	1.25	Additive	Bactericidal
	16	4	0.50	0.25	0.75	Additive	Bacteriostatic
	32	2	1.00	0.13	1.13	Additive	Bactericidal
	16	2	0.50	0.13	0.63	Additive	Bacteriostatic
	32	1	1.00	0.06	1.06	Additive	Bactericidal
	16	1	0.50	0.06	0.56	Additive	Bacteriostatic

**Table.5:** Combination effect of SBD and VAN on SA

Compound	Concentration (μg/mL)		FIC index		Sum FIC index	Interaction	Static/cidal effect
	SBD	OXA	SBD	OXA			
SBD2	8	4	1.00	1.00	2.00	Additive	Bacteriostatic
	4	4	0.50	1.00	1.50	Additive	Bacteriostatic
	2	4	0.25	1.00	1.25	Additive	Bacteriostatic
	1	4	0.13	1.00	1.13	Additive	Bacteriostatic
	0.5	4	0.06	1.00	1.06	Additive	Bacteriostatic
	8	2	1.00	0.50	1.50	Additive	Bacteriostatic
	4	2	0.50	0.50	1.00	Additive	Bacteriostatic
	8	1	1.00	0.25	1.25	Additive	Bacteriostatic
SBD4	16	4	0.50	1.00	1.50	Additive	Bacteriostatic
	8	4	0.25	1.00	1.25	Additive	Bacteriostatic
	4	4	0.13	1.00	1.13	Additive	Bacteriostatic
	2	4	0.06	1.00	1.06	Additive	Bacteriostatic
	16	1	0.50	0.25	0.75	Additive	Bacteriostatic

**Table.6:** Combination effect of SBD and VAN on MRSA

Compound	Concentration (μg/mL)		FIC index		Sum FIC index	Interaction	Static/cidal effect
	SBD	VAN	SBD	VAN			
SBD2	8	4	1.00	1.00	2.00	Additive	Bacteriostatic
	4	4	0.50	1.00	1.50	Additive	Bacteriostatic
	2	4	0.25	1.00	1.25	Additive	Bacteriostatic
	1	4	0.13	1.00	1.13	Additive	Bacteriostatic
	0.5	4	0.06	1.00	1.06	Additive	Bacteriostatic
	8	2	1.00	0.50	1.50	Additive	Bacteriostatic
	4	2	0.50	0.50	1.00	Additive	Bacteriostatic
	2	2	0.25	0.50	0.75	Additive	Bacteriostatic
	1	2	0.13	0.50	0.63	Additive	Bacteriostatic
	0.5	2	0.06	0.50	0.56	Additive	Bacteriostatic
	8	1	1.00	0.25	1.25	Additive	Bacteriostatic
	4	1	0.50	0.25	0.75	Additive	Bacteriostatic
	2	1	0.25	0.25	0.50	Synergy	Bacteriostatic
	1	1	0.13	0.25	0.38	Synergy	Bacteriostatic
	0.5	1	0.06	0.25	0.31	Synergy	Bacteriostatic
	8	0.5	1.00	0.13	1.13	Additive	Bacteriostatic
	8	0.25	1.00	0.06	1.06	Additive	Bacteriostatic
SBD4	32	4	1.00	1.00	2.00	Additive	Bactericidal
	16	4	0.50	1.00	1.50	Additive	Bacteriostatic
	8	4	0.25	1.00	1.25	Additive	Bacteriostatic
	4	4	0.13	1.00	1.13	Additive	Bacteriostatic
	2	4	0.06	1.00	1.06	Additive	Bacteriostatic
	32	2	1.00	0.50	1.50	Additive	Bacteriostatic
	16	2	0.50	0.50	1.00	Additive	Bacteriostatic
	8	2	0.25	0.50	0.75	Additive	Bacteriostatic
	4	2	0.13	0.50	0.63	Additive	Bacteriostatic
	2	2	0.06	0.50	0.56	Additive	Bacteriostatic
	32	1	1.00	0.25	1.25	Additive	Bacteriostatic
	16	1	0.50	0.25	0.75	Additive	Bacteriostatic
	32	0.5	1.00	0.13	1.13	Additive	Bacteriostatic
	16	0.5	0.50	0.13	0.63	Additive	Bacteriostatic
	8	0.5	0.25	0.13	0.38	Synergy	Bacteriostatic
	32	0.25	1.00	0.06	1.06	Additive	Bacteriostatic
	16	0.25	0.50	0.06	0.56	Additive	Bacteriostatic

### Anti-biofilm activity of SBD compounds and antibiotics against SA and MRSA cells in biofilm: individual and in combination

**Fig. 1:**
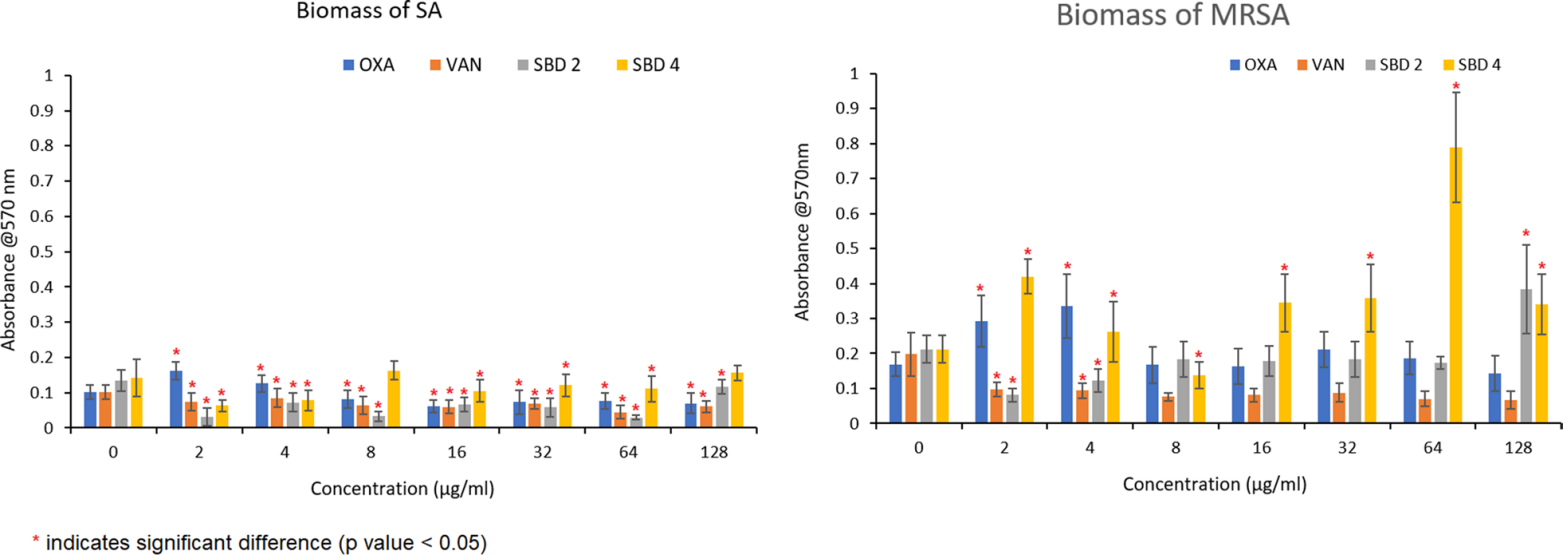
The effect of OXA, VAN, SBD2 and SBD4 on the biomass of SA and MRSA biofilms

**Fig. 2:**
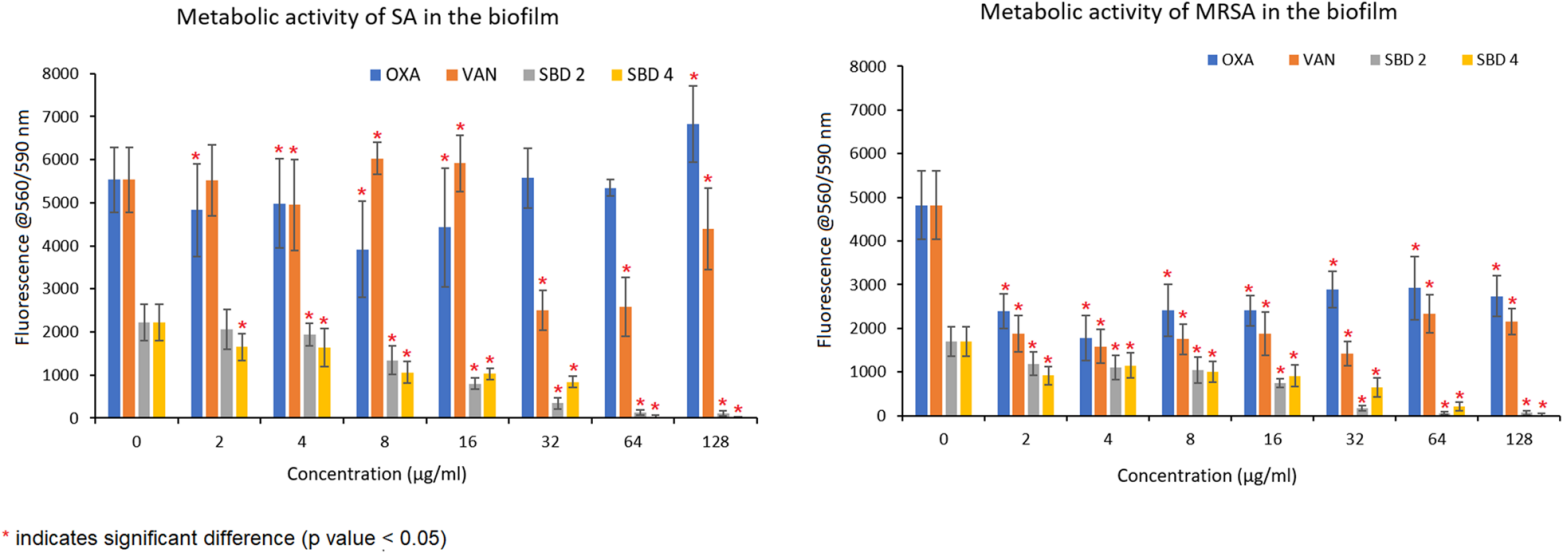
The effects of OXA, VAN, SBD2 and SBD4 on the metabolic activity of SA and MRSA cells in the biofilms

Exposure of pre-formed SA biofilms to SBD2, SBD4 and VAN showed significant decrease in the biomass in comparison to the control (no treatment) over a 24-h period, with the lowest readings at 2 µg/mL (Fig. 1). In contrast, OXA showed a significant increase in the biomass at 2 µg/mL and 4 µg/mL, followed by a significant decrease at 16 µg/mL. Hence, the MBEC values in SA were recorded as 2 μg/mL for VAN, SBD2 and SBD4, and 16 μg/mL for OXA as these concentrations were the lowest concentrations which showed significant reduction of biofilm in comparison to the control. The metabolic activity of SA cells in the biofilm treated with SBD2 and SBD4 was significantly decreased in all the concentrations tested. The lowest metabolic activity was recorded at 128 µg/mL. For OXA and VAN, significant reduction of the metabolic activity was observed at concentrations 8 to 16 µg/mL and 32 to 128 mg/mL, respectively (Fig. 2).

Similar to SA, the pre-formed MRSA biofilms showed significant reduction of biomass when treated with VAN, with the lowest readings at 2 µg/mL (Fig. 1). Exposure of the cells to OXA also seemed to promote the formation of biomass at concentrations below 4 µg/mL. SBD2 decreased the biomass at concentrations lower than 128 µg/mL, with the lowest reading at 2 µg/mL. SBD4 had also shown an increase in the biomass at all the concentrations tested, with the exception of 8 µg/mL. Hence, the MBEC values for VAN, SBD2 and SBD4 in MRSA were recorded as 2, 2 and 8 µg/mL, respectively. The metabolic activity of MRSA cells in the biofilm treated with OXA, VAN, SBD2 and SBD4 showed significant decrease in comparison to the control (Fig. 2).

The combinations of the SBD compounds with OXA or VAN at 1 × MBEC showed significant decrease in the biomass, indicating an additive interaction. Combinations of the compounds and antibiotics at concentrations lower than 1 × MBEC seemed to promote the formation of biomass. These findings showed that SBD2 and SBD4 were more effective when used individually against pre-formed MRSA biofilms.

### Cytotoxicity of SBD2 and SBD4

**Fig. 3:**
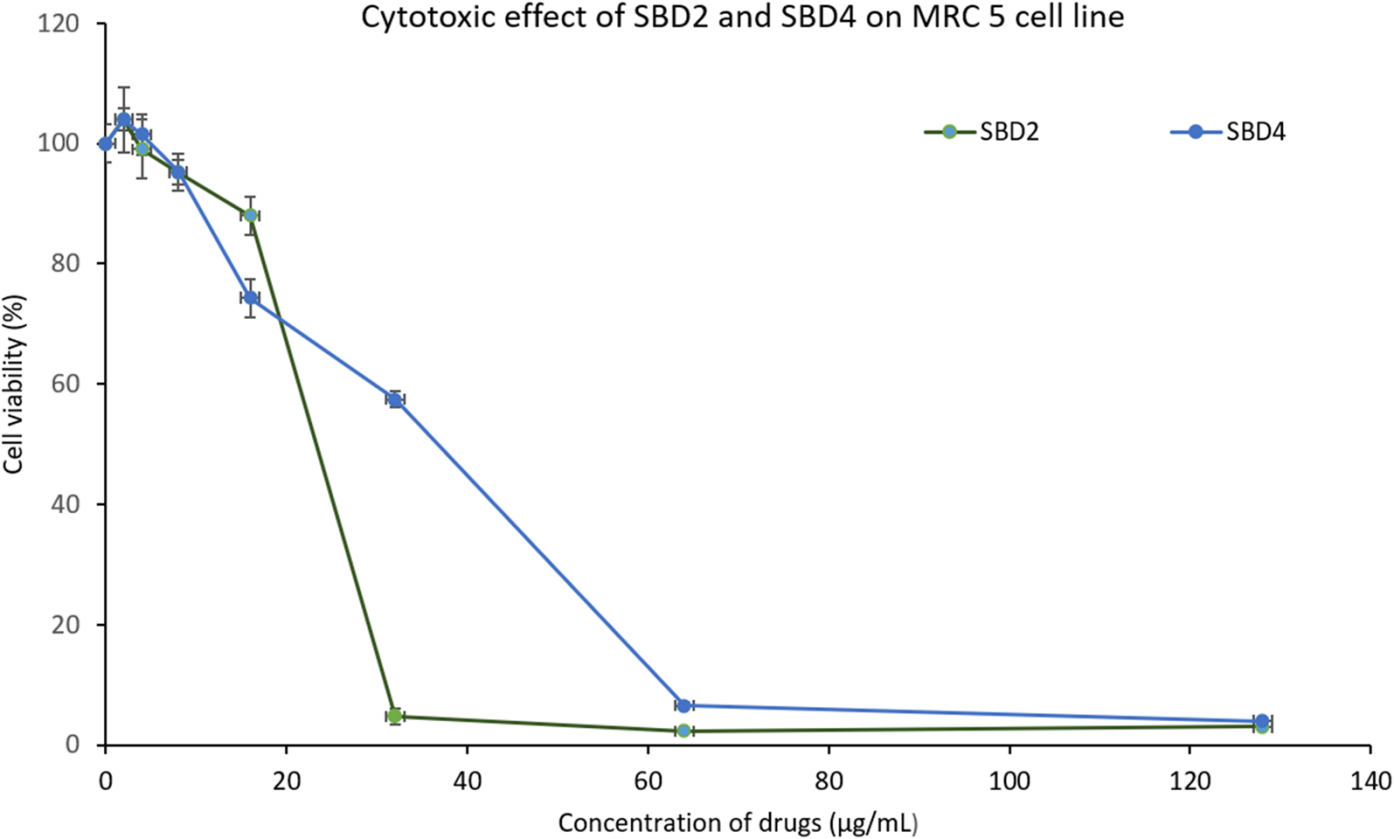
The cytotoxic effect of SBD2 and SBD4 on normal lung cells

The viability of the MRC5 cells decreased with the increase in the concentrations of the two compounds (Fig. 3). The IC_50_ and SI values of SBD2 and SBD4 are shown in Table 7. The SI values of more than 1 suggested that both the compounds were more cytotoxic to SA and MRSA than the MRC5 cells. Although the cytotoxic concentrations of the compounds were similar, SBD2 with a lower MIC value showed higher selectivity.

**Table.7:** Cytotoxicity of SBD2 and SBD4 on normal lung cells (MRC5) and their selectivity index against MRSA

Compound	IC_50_ (µg/ml)	Selectivity index
SBD2	45	5.63
SBD4	52	1.63

## Discussion

Methicillin-resistant *Staphylococcus aureus* (MRSA) has been constantly evolving and developing resistance against conventional antibiotics. One of the key features of MRSA that enables it to develop resistance to antibiotics and host immune system is its ability to form biofilm in indwelling medical devices. The shift from the planktonic to biofilm stages of growth in vitro involves multiple changes related to metabolism and the production of virulence factors. The initial cellular adherence to a surface is dependent on the synthesis of adhesive extracellular molecules. As the biofilm formation reached maturation stage, the biofilm consists of a variety of adhesive molecules and the metabolic activity of bacterial cells growing within the biofilm reaches a steady state. In response to the alteration in nutrient availability, oxygen fluctuations and increase of toxic products at this stage, these bacterial cells which are ‘trapped’ deep within the biofilm, alter their gene expression to promote transition from the sessile to the planktonic mode of growth. When cues that trigger the release of these planktonic cells are triggered, the sessile communities in the biofilm would then be dispersed to occupy new niches [12].

In this study, SBD2 and SBD4 showed enhanced inhibitory activities against the planktonic SA and MRSA, possibly due to complexation, improved permeability and increased molecular weight [13]. Other than *S. aureus*, studies have also shown that Schiff base copper complexes have enhanced antimicrobial ability against *Enterococcus faecalis*, *E. coli*, *Salmonella typhi*, *Klebsiella pneumoniae*, *P. aeruginosa* and *B.* subtilis [7, 14–16].

The ability of individual OXA, VAN, SBD2 and SBD4 to significantly reduce the biomass and/or metabolic activity of SA and MRSA in pre-formed biofilm were evaluated using the CV and RZ assays, respectively. CV non-specifically stains living and dead biomasses, and the matrix composed of extracellular polymeric substances. Therefore, CV is useful in the evaluation of the antibiofilm of a compound and an antibiotic in preventing biofilm formation and destroying preformed biofilms [17]. Metabolic activity, a common measure of cell viability, can be evaluated using oxidizable and reducible dyes such as resazurin dye (7-hydroxy-3H-phenoxazin-3-one 10-oxide). The reduction of resazurin correlates with the number of live cells [18].

Copper ion possesses redox properties where redox cycling that happens between Cu(II) and Cu(I) ion catalyzes the production of highly reactive hydroxyl radicals. This leads to the damage of DNA, denaturation of proteins, and deactivation of enzymes, lipids and other biomolecules in *S. aureus*. The redox reactions also produce O_2_ and H_2_O_2_ which possess oxidizing effects on vital *S. aureus* cell components such as lipoic acid [7]. The reduced Cu(I) complex may also inhibit DNA synthesis and adenosine triphosphate (ATP) production. Copper ions also showed high affinity with thiol and amino groups of *S. aureus* [19]. Thiol groups such as bacillithiol is a vital virulence factor contributing to pathogen fitness and conferring protection against host immune system, especially in MRSA. It was found that bacillithiol contributes to resistance during oxidative stress and detoxification of electrophiles, such as antibiotic Fosfomycin [20].

The mechanisms of action of the Cu(II) Schiff base complexes could also be postulated based on Overton’s concept of cell permeability [21] and the principles of chelation therapy [22]. According to Overton’s concept, lipid solubility of a molecule governs its entry into a cell, while the concept of chelation therapy suggests there is greater reduction in the polarity of the copper ion due to the overlap of the ligand orbital and partial sharing of the positive charge with donor groups [7, 22]. The copper ion with reduced polarity then increases the delocalization of π-electrons and enhances the lipophilicity of the complexes which leads to enhanced penetration of the Schiff base copper complexes into the lipid membrane. As a result, the metal binding sites in the bacterial enzymes are blocked and the enzymes deactivated. The cell wall permeability may be altered or damaged and the lipoproteins disorganised, further leading to interference of cell wall synthesis.

SBD2 and SBD4 have common structural properties that may have contributed to the antimicrobial effects, namely the azomethine group (C=N) that can form hydrogen bonds with active centre of cell constituents of *S. aureus* and interfere with protein synthesis. The presence of carboxylic moieties (COOH group) in the phenyl ring and the uncoordinated heteroatoms (N, S and O) were also believed to be able to bind with trace elements and denature the proteins in *S. aureus* [7].

Combinations between SBD2 and SBD4 with OXA and VAN were also found to be more effective than individual SBD2 and SBD4 against planktonic SA and MRSA cells. The compounds and antibiotics could act together against *S. aureus* with different mechanisms of action. For instance, OXA and VAN target the cell wall synthesis while the compounds may act on the DNA synthesis (or other examples of mechanisms of action of known antibacterials). The copper complex in the compounds could also increase the porosity of the bacterial cell wall which facilitates the entry of OXA and VAN into the bacteria. Hence, combinations of SBD and antibiotics could improve the antimicrobial effects against both susceptible and resistant strains of *S. aureus*.

Both SBD2 and SBD4 also showed promising anti-biofilm activities with significant reduction of biomass. Comparing the two compounds, SBD2 could be a better anti-biofilm agent as it could reduce the biomass over a wider range of concentrations with the least biomass at concentration of 2 µg/mL. Both the compounds have similar molecular structure (Table 7) with SBD4 having an additional hydroxyl (-OH) group. Studies have shown that the hydroxyl group in SBD can enhance the antimicrobial activity against a panel of bacteria and fungi [23, 24]. However, there is no evidence of anti-biofilm effects of this hydroxyl group on bacteria. SBD2 may act on certain essential components of the biofilm of MRSA such as eDNA, teichoic acids or other important proteins such as accumulation-associated protein (Aap) and extracellular matrix-binding protein (Embp) [25]. In addition, SBD2 may also act on the biosynthesis of an important biofilm component known as polysaccharide intercellular adhesin (PIA) in MRSA, as how sulfhydryl compounds strengthened certain metabolic pathways in the Embden–Meyerhof–Parnas pathway and pentose-phosphate pathway by repressing N-acetyl-glucosamine-associated polysaccharide metabolism [26].

The SI is an indirect measure of the therapeutic window, which can serve as a predictor of safety and efficacy of a compound during in vivo trials for a given bacterial infection [27]. The relatively high SI values of SBD2 and SBD4 infer that both the compounds were able to eliminate MRSA at concentrations below their cytotoxic concentrations. With a higher selectivity for MRSA, SBD2 has the potential to be further developed as an antimicrobial agent against infections caused by MRSA.

## Conclusion

To the best of our knowledge, this is the first report on the antimicrobial and antibiofilm activities of SBD2 and SBD4 as individual compounds and in combination with antibiotics against *S. aureus*. In summary, postulated mechanisms of action of both the compounds against *S. aureus* could include interference with cell wall synthesis, deactivation of cellular enzymes, denaturation of proteins in the organism, and formation of hydrogen bond through the azomethine group with the active centre of cell constituents in the organism. Further studies to elucidate the exact mechanisms of action of these compounds, particularly SBD2, on the planktonic cells and biofilm of MRSA could be carried out using the ‘omics’ technology. From the ‘omics’ profiles obtained, pathway characterization and identification of gene products as potential drug targets can be further explored.

## Supplementary Information

**Additional file 1: Fig. S1. **The biofilm formation profile of reference SA and MRSA over a duration of 24-h.

**Additional file 2: Table S1.** Preliminary study on the inhibition of oxacillin and vancomycin on SA and MRSA.

## Data Availability

All data relevant to the study are included in the article.

## Abbreviations

MRSA: Methicillin-resistant *Staphylococcus aureus*
VRSA: Vancomycin-resistant *Staphylococcus aureus*
SA: *Staphylococcus aureus*
OXA: Oxacillin
VAN: Vancomycin
SBD: Schiff base derivatives
DMSO: Dimethyl sulfoxide
MTT: 3-(4,5-Dimethylthiazole-2-yl)-2,5-diphenyltetrazolium bromide
CFU: Colony forming unit
RPMI: Roswell Park Memorial Institute Medium
MIC: Minimum inhibitory concentration
MBC: Minimum bactericidal concentration
MBEC: Minimum biofilm eradication concentration
FIC: Fractional inhibitory concentration
FBEC: Fractional biofilm eradication concentration
CV: Crystal violet
RZ: Resazurin
Aap: Accumulation-associated protein
Embp: Extracellular matrix-binding protein
PIA: Polysaccharide intercellular adhesion
