# Supplementary figures and images for: Antimicrobial and antibiofilm activities of Cu(II) Schiff base complexes against methicillin-susceptible and resistant Staphylococcus aureus
Source: Ann Clin Microbiol Antimicrob. 2021 Sep 24;20:67. doi: 10.1186/s12941-021-00473-4 (PMC8464119; doi:10.1186/s12941-021-00473-4)

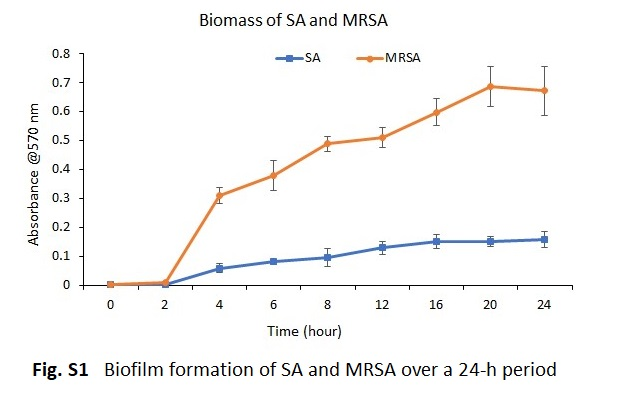

Supplement: Supplementary file 1 — Additional file 1: Fig. S1. The biofilm formation profile of reference SA and MRSA over a duration of 24-h. [file 12941_2021_473_MOESM1_ESM.png]
