# Supplementary material for: Antimicrobial and antibiofilm activities of Cu(II) Schiff base complexes against methicillin-susceptible and resistant Staphylococcus aureus
Source: Ann Clin Microbiol Antimicrob. 2021 Sep 24;20:67. doi: 10.1186/s12941-021-00473-4 (PMC8464119; doi:10.1186/s12941-021-00473-4)
Supplement: Supplementary file 2 — Additional file 2: Table S1. Preliminary study on the inhibition of oxacillin and vancomycin on SA and MRSA. [file 12941_2021_473_MOESM2_ESM.docx]

**Table S1.** Preliminary study on the inhibition of oxacillin and vancomycin on SA and MRSA

| Microorganism | OXA (µg/ml) | | VAN (µg/ml) | |
| --- | --- | --- | --- | --- |
|  | Test | CLSI [8] | Test | CLSI [8] |
| *S. aureus* ATCC 29213 | 2 | < 2 | 1 | < 2 |
| *S. aureus* ATCC 43300 | 8 | > 4 | 32 | > 16 |

Number of replicates, n=3×3
